# Reliability and validity of the German version of the Myositis Activities Profile (MAP) in patients with inflammatory myopathy

**DOI:** 10.1371/journal.pone.0217173

**Published:** 2019-06-03

**Authors:** Pierrette Baschung Pfister, Eling D. de Bruin, Caroline H. G. Bastiaenen, Britta Maurer, Ruud H. Knols

**Affiliations:** 1 Directorate of Research and Education, Physiotherapy Occupational Therapy Research Center, University Hospital Zurich, Zurich, Switzerland; 2 Department of Epidemiology, CAPHRI School for Public Health and Primary Care, Maastricht University, Maastricht, The Netherlands; 3 Department of Health Sciences and Technology, Institute of Human Movement Sciences and Sport, ETH Zurich, Zurich, Switzerland; 4 Division of Physiotherapy, Department of Neurobiology, Care Sciences and Society, Karolinska Institutet, Huddinge, Sweden; 5 Caphri Research Institute, Research Line Functioning and Rehabilitation, Department of Epidemiology, Maastricht University, Maastricht, The Netherlands; 6 Division of Rheumatology and Laboratory for Systemic Autoimmune Diseases, University Hospital of Zurich, Zurich, Switzerland; University of Lleida, SPAIN

## Abstract

The Myositis Activity Profile (MAP) is the only disease-specific questionnaire to assess limitations in activities of daily living (ADL) in patients with inflammatory myopathy (IM). Because a German version does not currently exist, this study’s aim was to translate the MAP and assess reliability and construct validity of the new version. Therefore, a cross-cultural adaptation was performed following international guidelines. Forty-eight patients with IM completed the German-MAP, twice within two weeks. They were also assessed using the Health Assessment Questionnaire (HAQ), 36-Item Short Form Survey (SF36), Manual Muscle Test (MMT8), Quantitative Muscle Testing (QMT) and Functional Index (FI-2). For discriminant validity, 48 age-and gender-matched healthy controls completed the German-MAP. Reliability was assessed using weighted kappa (K_w_). Correlations between the MAP and the HAQ, the physical (PCM) and mental (MCS) component scores of SF36 and the MMT8 and QMT muscle tests were assessed using Spearman correlation analysis. Discriminative validity was assessed by calculating the Area under the Curve (AUC). The German-MAP showed substantial reliability for the four subscales (K_w_: 0.65–0.71) and moderate to substantial reliability for the single items (K_w_: 0.57–0.77). The MAP showed good construct validity (high correlations with HAQ and PCM, moderate with FI-2, QMT and MMT8 and poor with MCS and pain) and acceptable discrimination for three subscales and two single items (AUC: 0.65–0.79). In conclusion, the German-MAP appears to be a reliable and valid questionnaire to assess ADL-limitations in patients with IM. Further research is required, both to substantiate these results and to evaluate responsiveness.

## Introduction

Inflammatory myopathies (IMs), including polymyositis (PM) and dermatomyositis (DM), are chronic, idiopathic inflammatory muscle diseases characterized by decreased muscle strength and endurance, general fatigue, and, in some cases, muscle pain and extra- muscular manifestations such as lung fibrosis [1]. Epidemiological studies have reported widely varying incidence rates between 0.1 and 7.89 cases per 100,000 annually [2–4]. Although relatively rare, IMs pose a significant socioeconomic burden causing increased medical costs and resource usage. Moreover, there is substantial disease morbidity [4, 5]. The most prominent clinical features in all subcategories of IMs are proximal and (often) symmetric muscle weakness and low muscle endurance, combined with progressive decline of functional performance over a period of weeks or months. This results in significant difficulties in everyday tasks, such as self-care or household chores, combined with limited community mobility [6, 7].

Until recently, activity limitation in patients with IM has been assessed with the Health Assessment Questionnaire (HAQ). Arthritis-specific instruments such as the HAQ permit comparisons between different rheumatologic diagnoses. However tools like this may be insufficiently sensitive to capture activity limitations caused by a specific non-arthritic condition [8]. The Myositis Activity Profile (MAP) was the first disease-specific activity limitation questionnaire for use with patients who have IM. It was developed in Sweden and later translated into the English language [8, 9]. Both the Swedish and English versions showed promising, preliminary psychometric properties in patients with IM. The MAP correlated well with the HAQ and showed fair-to-moderate correlation with muscle endurance (assessed with the functional index in myositis) and muscle strength (assessed with the manual muscle test) [8, 9]. To date, correlations between the MAP and health-related quality of life (HRQL) and pain have, to the best of our knowledge, not been determined. Because the MAP focusses on activity limitation and correlates with muscle endurance and strength, it can be hypothesized that the MAP tends rather to correlate with physical components of HRQL (e.g. with the physical component of the SF36 questionnaire), than with those components reflecting mental and pain aspects of life quality.

The MAP is based on the International Classification of Impairments, Disabilities and Handicaps-2 (ICIDH) Beta-2 draft, an earlier version of the International Classification of Functioning, Disability and Health (ICF). It includes 32 items, which are each answered on a seven-point Likert scale; where one equals no difficulty to perform and seven equals impossible to perform. These items are divided into four subscales (movement activities, activities of moving around, personal care, and domestic activities) and four single items (keeping in touch with close friends and relatives, avoiding overexertion during daily activities, being able to cope with work and/or housework to a satisfactory degree, and being able to do recreational activities of choice). The four subscales are scored using the median value of item responses within each, while the four single items are scored using the actual item response value. Additionally, a total median score across the four subscales and the four single items is also calculated [10]. Overall, the MAP assesses activity limitation within a broad perspective, meaning that patients weight their difficulties against their needs when answering the questions [11].

To date and to the best of our knowledge, a German version of the MAP has not been developed. Therefore, the aim of this study was to translate and cross-culturally adapt the MAP into German following international guidelines [12, 13], and then evaluate construct validity and reliability of this new version. We hypothesized that the total median score across the four subscales and the four single items of the German-MAP would correlate highly with the HAQ and the physical component of the SF36, moderately with muscle function (strength and endurance), but lower with pain and the mental component of the SF 36. Furthermore, we hypothesized acceptable discriminant validity, substantial reliability with no systematic variations between test and retest, no internal redundancy or poor internal consistency.

## Material and methods

### Participants

Patients with IM were recruited from the Department of Rheumatology at the University Hospital Zurich. Inclusion criteria were diagnosis of PM, DM or associated myositis, age over 18, and ability to read and understand German. Excluded were patients diagnosed with inclusion body myositis, osteoporosis, severe cardiovascular and/or pulmonary disease, pain syndrome, and paresis. To evaluate discriminative validity, a healthy control group individually matched by age and gender was recruited. Healthy was defined as not having any disease diagnosed by a medical doctor.

### Cross-cultural translation

The cross-cultural translation into German was performed in four steps, in accordance with international guidelines and with permission for translation from the original MAP developers [12, 13]. In step 1, two independent investigators each independently translated the MAP from English into German (T1 and T2). One of the translators was aware of the questionnaire concept. The second step was the synthesis of these two German versions, achieved by consensus discussion between the two translators. This produced a consensus German version (T1/2). In the third step, T1/2 was independently translated backwards by two native English-speaking persons, to produce two further English language versions (BT1 and BT2). Both persons were unaware of the questionnaire concept and were unfamiliar with the original English questionnaire. In step four, all four translators discussed any discrepancies retrieved between the original and the translated versions and produced a pre-final version. The understanding of this pre-final version was tested in practice with three patients with IM, before a final German version (S1 Appendix) was arrived at. This was then tested for validity and reliability.

### Assessments

The HAQ consists of 20 questions divided into eight sections: dressing, rising, eating, walking, hygiene, reaching, gripping objects, and performance of activities. Scoring within each section varies from zero (without any difficulty) to three (unable to do) [14].

The SF-36 is a generic patient-reported outcome measure aimed at quantifying health-related quality of life (HRQOL). The questionnaire includes 36 questions, divisible into a physical component summary (PCS) and a mental component summary (MCS). In both of these components, higher scores indicate better HRQOL [15].

The Functional Index in Myositis (FI-2) is a disease-specific observational tool to measure muscle endurance. The FI-2 measures the number of repetitions performed in seven muscle groups/activities on the dominant side of the body. Each muscle group is scored by the number of correctly performed repetitions, ranging from 0–60 for shoulder flexion, shoulder abduction, neck flexion, hip flexion, knee extension and step test, and from 0–120 for heel and toe lifts. Sixty and 120 repetitions respectively indicate normal endurance [16]. To compute a total percentage score for all performed repetitions, all individual muscle group scores are summed and then divided by seven.

The MMT8 is a disease specific subset of eight proximal, distal, and axial muscle groups tested unilaterally to measure muscle strength. Each muscle group is scored from 0–10 on the Kendall scale. Zero indicates no muscle contraction and ten equals normal strength. The scores are summed up to a total score with a range varying from 0–80 [17].

Quantitative Muscles Testing (QMT) was performed with the microFET2. Maximum muscle strength of each of the muscle groups included in the MMT8 (shoulder abduction, elbow flexion, ankle extension, hip abduction, hip extension, knee extension, wrist extension, neck flexion) was tested on a continuous scale (Newton). To compute a total score, values of each muscle group were summated and this sum was then divided by eight.

Pain was measured using a Visual Analog Scale (VAS). A ten centimetres horizontal line with the anchors ‘no pain’ (score of zero) and ‘worst pain’ (score of 100) was used [18].

### Experimental procedures

Patients attended the university hospital Zurich on two occasions, to provide data to test construct validity and reliability. During the first visit, patients’ demographic data were collected, questionnaires (MAP, HAQ, SF36, pain) were completed and strength assessments (MMT8, QMT, FI-2) were performed by one of two experienced physiotherapists. The latter had both received instruction and training prior to the investigation. All patients, whose health status remained unchanged, received a further copy of the MAP at their second visit. This was to provide data for reliability assessment. The time interval between the two visits ranged from one to two weeks. Health status was assessed with the question: did your health status change since the last visit to the hospital? The healthy control group individuals each received a copy of the MAP by post at home and were requested to complete and return it via registered post-paid mail envelope.

### Data analysis

As the MAP uses an ordinal scale, nonparametric statistical analysis was used. Data are presented as median value with lower and upper quartiles and analyzed accordingly. Analysis was performed using SPSS version 23.0.

#### Construct validity

Convergent validity was assessed by determining the Spearman correlation coefficient for the relationship between the median scores of subscales and single items of the German MAP and the HAQ, the physical and mental component score of the SF36, the pain scale, the FI-2, the MMT8 and the QMT. A Spearman correlation coefficient of 0–0.25 was considered as no or very low correlation, 0.26–0.4 as low correlation, 0.41–0.69 as moderate correlation, 0.70–0.89 as high and 0.90–1.0 as very high correlation [19].

The discriminative value of the MAP based on the gold standard (healthy by self-report) was assessed by plotting the receiver operating characteristic (ROC) curve and by calculating the area under the curve (AUC). The greater the area under the curve, the more accurate the test is. We used the following classification proposed by Hosmer and Lemeshow: AUC = 0.5: no discrimination, 0.7 to < 0.8: acceptable discrimination, 0.8 to 0.9: excellent discrimination and > 0.9 outstanding discrimination [20].

#### Reliability

To determine reliability, the weighted kappa coefficients (K_W_) of subscales and the single items were computed using GraphPad software (http://faculty.vassar.edu/lowry/kappa.html). To interpret kappa values we applied the classification from Landis and Koch (>0.8: almost perfect, 0.61–0.8: substantial, 0.41–0.6: moderate, 0.21–0.4: fair, <0.2 slight) [21]. The Mann-Whitney-U test was used to analyse systematic variations between test and retest of the MAP. The level of significance was set to p≤0.05.

Based on the evaluation of the original Swedish version, internal redundancy and internal consistency were analyzed [8]. For internal redundancy, correlations between questions in the subscales and for internal consistency correlations between individual items and their subscales, after the actual item had been removed from the score, were computed. Spearman correlation coefficients (r_s_) >0.90 were considered to indicate internal redundancy, and r_s_ <0.50 to indicate poor internal consistency [8].

Additionally, Cronbach`s alpha coefficient was calculated to evaluate item fit within subscales. Cronbach`s Alpha values between 0.7 and 0.9 were considered as acceptable [22].

The study complied with the Helsinki Declaration and was approved by the local ethics committee (registration no. 2014–0022 of the Cantonal Zurich Ethical Committee). All participants signed an informed consent.

## Results

**Fig 1:**
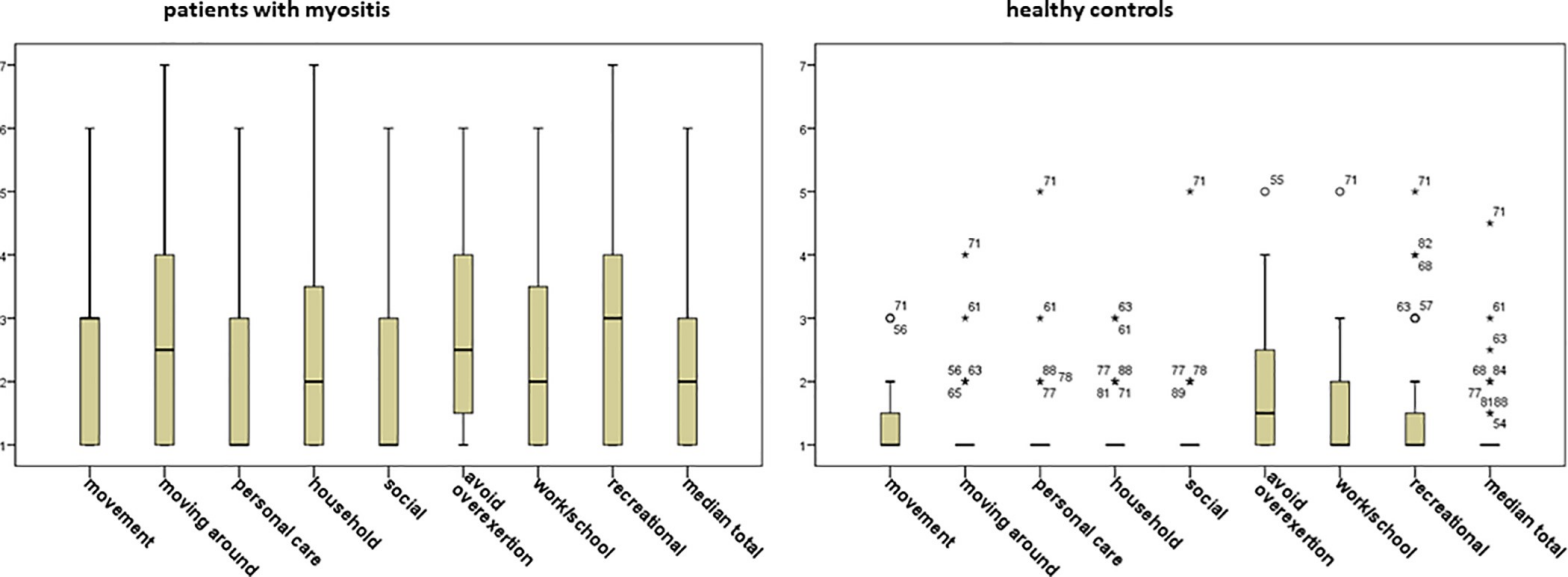
Score distribution of the subscales and single items of the Myositis Activity Profile in patients with IM and healthy controls, 1 = no difficulty, 7 = impossible to do.

From the 76 patients invited to participate in this study, 28 declined because of “lack of time”, “bad health condition” or “no interest”. Baseline characteristics of the 48 patients and 48 healthy controls are summarized in Table 1. Healthy controls had a significant lower BMI than patients (median of 23 versus 26, p-value < 0.01). Score distribution of the subscales and single items of the MAP are shown in Fig 1.

**Table 1:** Baseline characteristics.

	patients with IM	healthy controls
	N:48	N: 48
Sex, n (%)		
female	36 (75)	36 (75)
male	12 (25)	12 (25)
Age, in year		
median (Q1-Q3)	60 (51–66)	60 (51–66)
BMI, in kg/cm^2^		
median (Q1-Q3)	26 (22–29)	23 (20–25)
Diagnosis, n (%)		
PM	17 (35.4)	
DM	20 (41.7)	
Associated	11 (22.9)	
Disease stage, n (%)		
acute	9 (18.8)	
subacute	7 (14.6)	
chronic	32 (66.7)	
Time since diagnosis (months)		
median (Q1-Q3)	18 (3–54)	
HAQ, 0.00–3.00		
median (Q1-Q3)	0.81 (0.13–1.38)	
pain, VAS, 0–100		
median (Q1-Q3)	14 (0–31)	
SF36		
PCS		
median (Q1-Q3)	35 (30–49)	
MCS		
median (Q1-Q3)	51 (42–58)	
FI-2		
hip flexion		
median (Q1-Q3)	13 (5–27)	
shoulder flexion		
median (Q1-Q3)	22 (10–55)	
total score		
median (Q1-Q3)	25 (16–46)	
MMT8 total score		
median (Q1-Q3)	71 (67–76)	
QMT total score		
median (Q1-Q3)	106 (91–137)	

PM: polymyositis, DM: dermatomyositis, FI-2: Functional Index 2, HAQ: Health Assessment Questionnaire, PCS: physical component score, MCS: mental component score, Q1: lower quartile, Q3: upper Quartile

### Construct validity

Convergent validity: The median of subscales and single items of the German MAP correlated highly with the HAQ and the physical component score of the SF36; moderately with the total scores of the FI-2, the QMT and the MMT8; and poorly with the mental component score of the SF36 and with pain scale scoring (Table 2). Correlation coefficient values between the MAP and the SF36 (PCS and MCS), the MMT8 and the FI-2 were all negative, as these are all inverse relationships.

**Table 2:** Correlations between the total median score across the four subscales and the four single items of the German Myositis Activity Profile (MAP) and other measures.

Measure	German MAP r_s_	CI
Health Questionnaires, N:46		
HAQ	0.77	0.60–0.91
SF 36: PCS	-0.78	-0.87 - -0.63
SF 36: MCS	-0.28	-0.55–0.27
Muscle strength, N: 47		
MMT8	-0.59	-0.76 - -0.34
QMT_total score	-0.59	-0.76 - -0.34
Muscle endurance, N:45		
FI-2 total score	-0.64	-0.44 - -0.78
pain (during last week), N: 46	0.38	0.10–0.62

rs: Spearman correlation coefficient; MMT8: Manuel Muscle Test; FI-2: Functional Index 2; HAQ: Health Assessment Questionnaire

**Fig 2:**
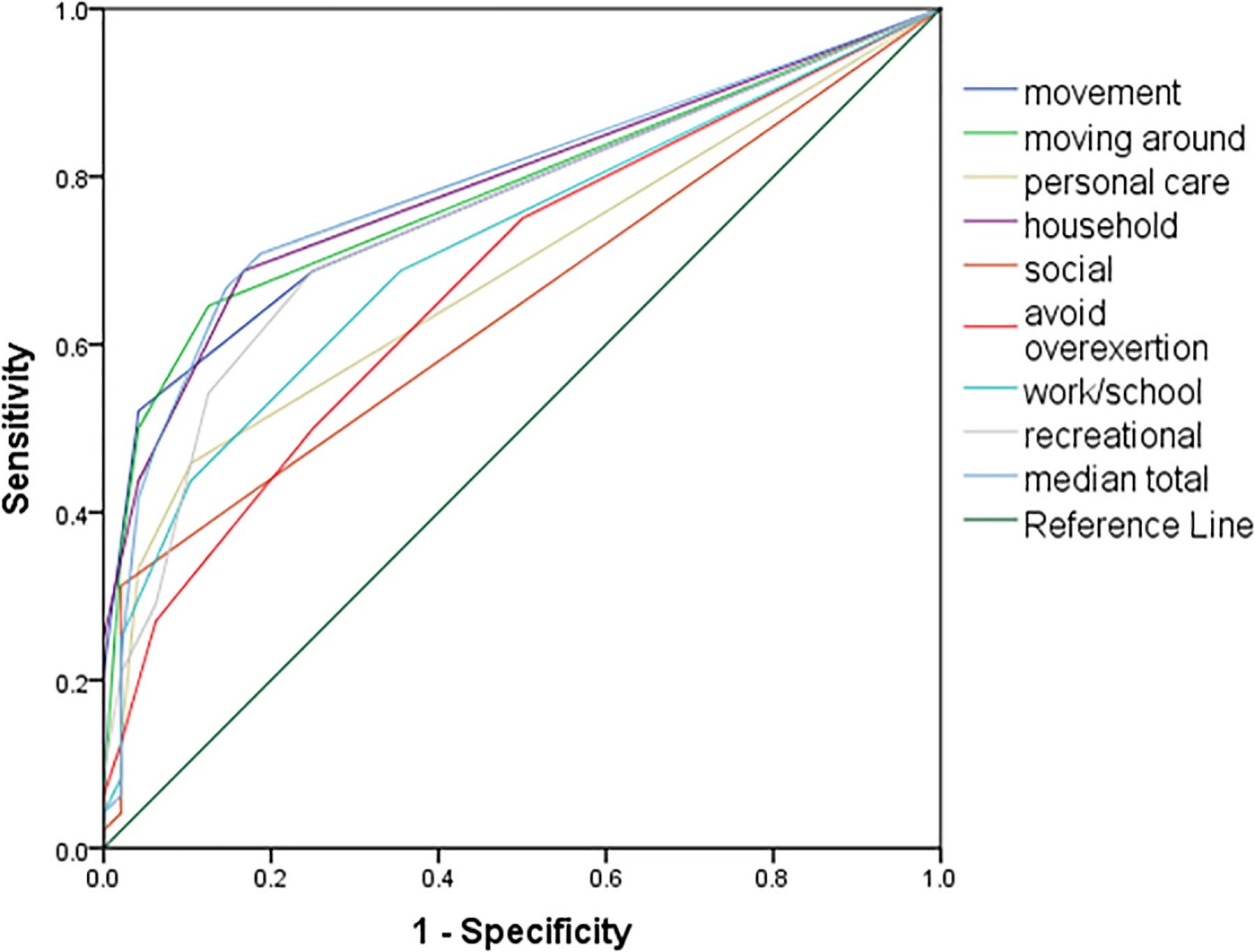
Receiver operating characteristic (ROC) curve.

Discriminative validity: The ROC curves are shown in Fig 2. Three out of four subscales and two out of four single items achieved acceptable AUC values (0.72–0.79). Subscale “personal care” and single questions “social” and “avoid over-exertion” indicated no acceptable discrimination (AUC between 0.65 and 0.69), as illustrated in Table 3. Specificity and Sensitivity of possible cut-off values are presented in Table 4. For the subscales/single items a cutoff level of two points and for the total median a cutoff value of 1.5 points leads to highest sensitivity. When considering the specificity, cutoff levels of 3 points for the subscales/single item values and 3.5 points for the total median values are therefore ideally required.

**Table 3:** Area under the curve.

German MAP	AUC	CI
Subscales		
Movement	0.78	0.68–0.87
Moving around	0.78	0.69–0.88
Personal Care	0.69	0.58–0.79
Domestic	0.79	0.70–0.88
Single Items		
Social	0.65	0.54–0.76
Avoid overexertion	0.68	0.57–0.79
Work/school	0.72	0.62–0.82
Recreational	0.75	0.66–0.85

MAP: Myositis Activity Profile; AUC: Area under the Curve, CI: Confidence Interval

**Table 4:** Sensitivity, specifity at different cut-offs.

German MAP	cut-off	Sensitivity	Specificity
Subscales			
Movement	2.00	68.75	75.00
	3.00	52.08	95.83
Moving around	2.00	64.58	87.50
	3.00	50.00	95.83
Personal care	2.00	45.83	89.58
	3.00	33.33	95.83
Household	2.00	68.75	83.33
	3.00	43.75	95.83
Single Items			
Social	2.00	41.67	83.33
	3.00	31.25	97.92
Avoid overexertion	2.00	75.00	50.00
	3.00	50.00	75.00
Work/school	2.00	68.75	64.58
	3.00	43.75	89.58
Recreational	2.00	68.75	75.00
	3.00	54.17	87.50
Total median	1.50	70.83	81.25
	2.00	66.67	85.42
	0.50	47.92	93.75
	3.00	41.67	95.83
	3.50	22.92	97.92
	4.00	18.75	97.92
** **	4.50	6.25	97.92

MAP: Myositis Activity Profile

### Reliability

Forty-four patients declared no change in their health status and were, therefore, included in the reliability analysis. Weighted kappa coefficient values ranged from 0.65 to 0.71 for the MAP subscales and 0.57 to 0.77 for the single items. No systematic differences between the two test occasions were recorded (Table 5).

**Table 5:** Test retest reliability of subscales and single items of the German MAP.

MAP	Test Median (Q1-Q3) N: 44	Retest Median (Q1-Q3) N: 44	K_w_	95%CI	Sign Test p
Subscales					
Movement	3.3 (1.0–3.0)	2.0 (1.0–4.0)	0.67	0.54–0.81	0.34
Moving around	2.5 (1.0–4.0)	2.0 (1.0–3.0)	0.65	0.54–0.75	0.09
Personal Care	1.0 (1.0–3.0)	1.0 (1.0–3.0)	0.7	0.55–0.84	0.31
Domestic	2.0 (1.0–3.8)	3.0 (1.0–4.0)	0.71	0.61–0.82	0.78
Single Items					
Social	1.0 (1.0–3.0)	1.0 (1.0–3.0)	0.77	0.63–0.92	0.57
Avoid overexertion	2.5 (1.3–4.0)	2.5 (1.0–4.0)	0.57	0.41–0.74	0.35
Work/school	2.0 (1.0–3.8)	2.5 (1.0–4.0)	0.67	0.53–0.81	0.2
Recreational	3.0 (1.0–4.0)	3.0 (1.0–4.0)	0.71	0.55–0.87	0.27

MAP: Myositis Activity Profile; Q1: lower quartile, Q3: upper Quartile; K_W_: weighted kappa coefficient; CI: Confidence Interval

Internal redundancy: Spearman Correlation Coefficients between items in the subscale “movement activities” (n: 9) varied between 0.29 and 0.81, in the subscale “moving around” (n: 4) between 0.60 and 0.76, in the subscale “personal care” (n: 9) between 0.33 and 0.91, and in the subscale “household” (n: 6) between 0.41 and 0.88. The two questions about “washing and combing hair” were considered redundant (r_s_ = 0.91).

Internal consistency: Correlations between individual items and their subscales after the actual item had been removed from the score ranged from r_s_ = 0.45 to 0.83 for the subscale “movement activities”, r_s_ = 0.70 to 0.78 for the subscale “moving around”, r_s_ = 0.45 to 0.76 for the subscale “personal care”, and r_s_ = 0.48 to 0.87 for the subscale “household”. Three items (standing for longer period, wiping yourself after bathroom, putting plates and glasses away) indicate poor consistency.

Cronbach`s alpha coefficient values for the four subscales ranged between 0.89 and 0.94 (movement: 0.94, moving around: 0.89, personal care: 0.94, household and 0.93).

## Discussion

The German-MAP is the first disease-specific questionnaire for assessing limitations in activities of daily living in German-speaking patients with IM. This new German version of the MAP revealed acceptable construct validity: the majority of our hypotheses for discriminative validity were confirmed. Good test retest reliability was recorded. Kappa values for all subscales and three out of the four single items were substantial. The single question “avoid over-exertion” just failed to reach substantial reliability. Cronbach`s Alpha was acceptable for the subscale “moving around”. For the three other subscales Cronbach`s Alpha was higher than expected.

Our results for the convergent validity are in line with previous studies. All three versions of the MAP demonstrated higher correlations with a generic activity limitation questionnaire (HAQ) than with muscle function assessment scores. The correlations between the two questionnaires (MAP and HAQ) were similar in all three countries (Spearman correlation coefficients ranging between 0.69 and 0.77). For muscle function, the correlations were more heterogeneous across the different language versions. While the correlation coefficients of the MAP with muscle endurance in the Swedish and German cohorts were moderate (0.55 and 0.64), those in the English cohort were lower and poorer (between 0.29 and 0.47). Correlations between the MAP and muscle strength were solely determined in the English and German cohorts. Also for muscle strength, the English cohort showed lower correlations with the MAP compared to the German cohort (0.35 versus 0.59, respectively). Reasons for these lower correlations between the MAP and muscle function in the English cohort are unclear. The subject sample in the German cohort is slightly older than the two other cohorts (60 years versus 55 and 52 years), however, the Swedish cohort has the highest MAP scores. Concerning muscle strength, the English and German cohorts were comparable [8, 9]. Future studies with larger samples may shed greater light on this issue. The current correlation is an estimate and only with increasing sample size can we hope to progress towards a more stable and representative value [23].

In general, muscle strength and muscle endurance values correlated only moderately with the MAP. This emphasizes the importance of using Health-Related Patient-Reported Outcomes as well as assessments of physical performance. This was the first time that the MAP has been compared with the SF36 and with pain scores. The higher correlation found with the physical component summary than with the mental component summary of the SF36 supports the notion that the MAP does measure activity limitation. Although pain is an important outcome measure in patients with IM, it did not seem to affect activity limitation in our sample. The reason for this could be that our sample reported rather low pain levels on the VAS.

The gender and age matched control group indicated lower scores in all subscales/single items when compared with patients (Fig 1). Nevertheless, three items did not reach acceptable discriminative validity. One of these items was “avoid overexertion”. This is the only item that has a median higher than one in the healthy control group. Avoid overexertion does not seem to be an illness-related problem, but rather an individual or age-related challenge. Although our controls had to be healthy (by self-report), it is not impossible that they suffered from age-related problems such as physical deconditioning or arthritis, especially as half of our sample was older than 60. The remaining two items with low discriminative validity were “personal care” and “social”. These were the two items with the lowest rating in our patients group (median of one) as well as in the American and Swedish cohort [8, 9]. Patients seem to manage their personal care despite physical limitations. Perhaps this is because they have developed good coping strategies and/or have adapted their personal environments in such a way that they can live independently. For example, one woman in our sample told us that because she had problems drying her long hair she had it cut short. The item “social” is defined as “to keep in touch with and meeting friends or relatives”. It appears that, despite their physical limitations, the social lives of people who have IM are not strongly affected.

The level at which the cut-off value should be set for discrimination between patients and healthy persons is not obvious. When considering only the sensitivity, the cutoff should be at 2 points, but when considering the specificity, the cutoff would be at 3 points. Therefore, it is questionable if the MAP should be used as a screening tool. In actuality, the MAP was developed for assessing activity limitation among patients with IM [8], rather than a discriminatory tool.

Assessment items “washing hair” and “combing hair” both demonstrated high internal redundancy. In fact, these two activities are very similar. Because the item “washing hair” is rated lower, it could potentially be excluded from the questionnaire. In three out of the four subscales, one item indicated poor internal consistency: “standing for long period” from the subscale “movement”, “wiping yourself after bathroom” from the subscale “personal care” and “putting plates and glasses away” from the subscale “household”. For the items “standing for a longer period” and “putting plates and glasses away” it is questionable whether they belong to their current subscales. “Standing for a longer period” is the only item in the subscale “movement” that is a static rather than a dynamic activity. All the other activities are dynamic ones. This may be a reason why this item does not appear to fit this subscale. In the subscale “household” all activities other than “putting plates and glasses away” demand predominantly lower rather than upper extremity performance, or else focus on the body as a whole. Because the activity involves putting plates and glasses away in the top of a cupboard, other attributes such as adequate upper limb strength and range of motion are required.

As might be anticipated, our study had certain strengths as well as limitations. One strength is that we were almost able to recruit the COSMIN initiative minimum recommended number of 50 participants [24], despite IM being a relatively rare disease. Sixty-three percent of the patients, who visited our department and who were informed about this study, satisfied our selection criteria and agreed to participate. Another strength is the inclusion of a healthy control group. This is the first time that the MAP has been compared across both patients and healthy individuals. A limitation may be that the healthy controls and patients were only matched by age and gender and not by other possibly biasing factors; e.g. their physical activity level. The healthy control group had a significant lower BMI than patients; this may be an indication that the former were more active than the latter. Therefore, we do not know if healthy controls have fewer activity limitations than patients because they are more active, or if they are more active because they do not have muscle symptoms. To definitively exclude the influence of physical activity, its level across both patients and healthy controls should ideally be controlled for. Another possible limitation is that the study was performed in the German speaking part of Switzerland. To avoid a Swiss bias to the translation, one translator had a German background. Nevertheless, participants from other German speaking countries, such as Germany or Austria, might have judged certain items differently. A further limitation of our study is that we could not conduct a factor analysis. For this analytical approach, a minimum of four to ten cases per item is required [22], a participant number that was beyond our project’s capacity. Based on the encouraging findings of our study an international multi-center study might be considered to assess the replicability of our findings.

Although the MAP showed acceptable validity, there are some ambiguities concerning the structure and scoring of this questionnaire, which should also be considered in any future studies. The score of a subscale is the median of the scores from all items within it. Because the median is unaffected by extreme scores in such a scoring strategy, easier items have greater influence in the scoring of a subscale. This leads to the fact that all patients, who have no difficulty in more than fifty percent of the activities of a subscale end up having the same score, independently of the scoring of the other items. This scoring approach may lead to underestimation of the activity limitation of participants. Another pitfall of the MAP could be the fact that patients have to weight their difficulties against their needs when answering questions. Patients‘ abilities to reliably weight both the importance and the difficulty of their own performance has yet to be investigated [8].

This initial validation of the German version of the MAP revealed acceptable validity and good reliability for assessing activity limitation among German speaking Swiss patients with IM. Further validation is required to fully verify these results, using German and/or Austrian participant groups. Furthermore, structure and scoring as well as responsiveness of the MAP should ideally be evaluated in a larger multicenter study.

## Supporting information

S1 Appendix German-MAP. (PDF)

S1 Dataset MAP healthy. (XLSX)

S2 Dataset MAP patients. (XLSX)

## Abbreviations

AUC: Area Under the Curve
DM: Dermatomyositis
FI-2: Functional Index in Myositis
HAQ: Stanford Health Assessment Questionnaire Disability Index
HRQOL: Health Related Quality of Life
IM: Inflammatory Myopathy
K_W_: weighted Kappa coefficients
MAP: Myositis Activity Profile
MCS: Mental Component Summary
MMT: Manuel Muscle Test
PM: Polymyositis
PCS: Physical Component Summary
QMT: Quantitative Muscles Testing
ROC: Receiver Operating Characteristic
r_s_: Spearman correlation coefficients
VAS: Visual Analog Scale
