# Supplementary material for: Reliability and validity of the German version of the Myositis Activities Profile (MAP) in patients with inflammatory myopathy
Source: PLoS One. 2019 Jun 3;14(6):e0217173. doi: 10.1371/journal.pone.0217173 (PMC6546213; doi:10.1371/journal.pone.0217173)
Supplement: S1 Appendix — (PDF) [file pone.0217173.s001.pdf]

# Myositis-Aktivitätenprofil (MAP)

## (Fragebogen über tägliche Aktivitäten)

(adaptiert nach Alexanderson H et al. The myositis activity profile – Initial validation for assessment of polymyositis/dermatomyositis in the USAR. J Rheumatol 2012;39:11; Alexanderson H et al. Development of the myositis activities profile – validity and reliability of a self-administered questionnaire to assess activity limitations in patients with polymyositis/dermatomyositis. J Rheumatol 2002;29:11)

Bitte beantworten Sie die folgenden Fragen über Ihre tägliche Aktivitäten. Kreuzen sie das Feld an, bei dem Sie das Gefühl haben, dass es Ihre Erfahrungen der letzten sieben Tagen am besten beschreibt.

„**Schwierigkeiten**“ beinhaltet dabei sowohl wie anstrenged es für Sie war, eine Aktivität auszuüben, als auch wie wichtig es für Sie war, die beschriebene Aktivität ausführen zu können. Versuchen Sie Ihre Schwierigkeiten mit Ihren Bedürfnissen zu gewichten, wenn Sie die Fragen beantworten.

Beispiel 1: Sie haben grosse Mühe in die Hocke zu gehen (und Aktivitäten auszuführen), aber Sie mussten dies in der letzten Woche nicht tun. Daher können Sie diese Aktivität als wenig schwierig einstufen.

Beispiel 2: Sie haben geringe Mühe beim Kochen, jedoch mussten/wollten Sie in der letzten Woche mehrmals täglich kochen. Daher können Sie diese Aktiviät als sehr schwierig einstufen.

Beantworten Sie nun bitte **alle folgenden Fragen** mit einer bestmöglichen Einschätzung und kreuzen Sie jeweils nur ein Feld pro Frage an.

## Bewegung

**1) Wie schwierig ist es für Sie in Ihrem Alltag während längerer Zeit zu stehen (z.B. in einer Schlange anzustehen)?**

| (1) | (2) | (3) | (4) | (5) | (6) | (7) |
|---|---|---|---|---|---|---|
| ☐ | ☐ | ☐ | ☐ | ☐ | ☐ | ☐ |
| Gar nicht schwierig | sehr wenig schwierig | wenig schwierig | mässig schwierig | sehr schwierig | extrem schwierig | unmöglich |

**2) Wie schwierig ist es für Sie in Ihrem Alltag beim Vordersitz eines Autos ein bzw- auszusteigen?**

| (1) | (2) | (3) | (4) | (5) | (6) | (7) |
|---|---|---|---|---|---|---|
| ☐ | ☐ | ☐ | ☐ | ☐ | ☐ | ☐ |
| Gar nicht schwierig | sehr wenig schwierig | wenig schwierig | mässig schwierig | sehr schwierig | extrem schwierig | unmöglich |

**3) Wie schwierig ist es für Sie in Ihrem Alltag einen Gegenstand vom Boden aufzuheben (zum Beispiel etwas vom Boden auf den Tisch zu heben)?**

| (1) | (2) | (3) | (4) | (5) | (6) | (7) |
|---|---|---|---|---|---|---|
| ☐ | ☐ | ☐ | ☐ | ☐ | ☐ | ☐ |
| Gar nicht schwierig | sehr wenig schwierig | wenig schwierig | mässig schwierig | sehr schwierig | extrem schwierig | unmöglich |

**4) Wie schwierig ist es für Sie in Ihrem Alltag eine Umhängetasche oder einen Rucksack mit mehr als einem Kilo Gewicht zu tragen?**

| (1) | (2) | (3) | (4) | (5) | (6) | (7) |
|---|---|---|---|---|---|---|
| ☐ | ☐ | ☐ | ☐ | ☐ | ☐ | ☐ |
| Gar nicht schwierig | sehr wenig schwierig | wenig schwierig | mässig schwierig | sehr schwierig | extrem schwierig | unmöglich |

**5) Wie schwierig ist es für Sie in Ihrem Alltag eine volle Einkaufstasche vom Ladentisch oder vom Auto auf den Boden zu stellen?**

| (1) | (2) | (3) | (4) | (5) | (6) | (7) |
|---|---|---|---|---|---|---|
| ☐ | ☐ | ☐ | ☐ | ☐ | ☐ | ☐ |
| Gar nicht schwierig | sehr wenig schwierig | wenig schwierig | mässig schwierig | sehr schwierig | extrem schwierig | unmöglich |

**6) Wie schwierig ist es für Sie in Ihrem Alltag ein Kind, Haustier oder Gegenstände in ihre Armen hoch zu heben?**

| (1) | (2) | (3) | (4) | (5) | (6) | (7) |
|---|---|---|---|---|---|---|
| ☐ | ☐ | ☐ | ☐ | ☐ | ☐ | ☐ |
| Gar nicht schwierig | sehr wenig schwierig | wenig schwierig | mässig schwierig | sehr schwierig | extrem schwierig | unmöglich |

**7) Wie schwierig ist es für Sie in Ihrem Alltag Gegenstände mit einer oder beiden Händen zu greifen (beispielsweise eine Bratpfanne oder einen Hammer)?**

| (1) | (2) | (3) | (4) | (5) | (6) | (7) |
|---|---|---|---|---|---|---|
| ☐ | ☐ | ☐ | ☐ | ☐ | ☐ | ☐ |
| Gar nicht schwierig | sehr wenig schwierig | wenig schwierig | mässig schwierig | sehr schwierig | extrem schwierig | unmöglich |

**8) Wie schwierig ist es für Sie in Ihrem Alltag Konfitürengläser, Frischhaltedosen (z.B. Tupperware) oder Verpackungen zu öffnen?**

| (1) | (2) | (3) | (4) | (5) | (6) | (7) |
|---|---|---|---|---|---|---|
| ☐ | ☐ | ☐ | ☐ | ☐ | ☐ | ☐ |
| Gar nicht schwierig | sehr wenig schwierig | wenig schwierig | mässig schwierig | sehr schwierig | extrem schwierig | unmöglich |

**9) Wie schwierig ist es für sie für Sie in Ihrem Alltag eine schwere Türe zu öffnen (beispielsweise eine Auto-, Restaurant-, oder Ladentüre)?**

| (1) | (2) | (3) | (4) | (5) | (6) | (7) |
|---|---|---|---|---|---|---|
| ☐ | ☐ | ☐ | ☐ | ☐ | ☐ | ☐ |
| Gar nicht schwierig | sehr wenig schwierig | wenig schwierig | mässig schwierig | sehr schwierig | extrem schwierig | unmöglich |

## Aktivitäten der Fortbewegung

**10) Wie schwierig ist es für Sie in Ihrem Alltag mehr als 800 Meter auf einem ebenen Boden zu gehen?**

| (1) | (2) | (3) | (4) | (5) | (6) | (7) |
|---|---|---|---|---|---|---|
| ☐ | ☐ | ☐ | ☐ | ☐ | ☐ | ☐ |
| Gar nicht schwierig | sehr wenig schwierig | wenig schwierig | mässig schwierig | sehr schwierig | extrem schwierig | unmöglich |

**11) Wie schwierig ist es für Sie in Ihrem Alltag zu rennen (beispielsweise auf einen Bus zu rennen oder zu joggen)?**

| (1) | (2) | (3) | (4) | (5) | (6) | (7) |
|---|---|---|---|---|---|---|
| ☐ | ☐ | ☐ | ☐ | ☐ | ☐ | ☐ |
| Gar nicht schwierig | sehr wenig schwierig | wenig schwierig | mässig schwierig | sehr schwierig | extrem schwierig | unmöglich |

**12) Wie schwierig ist es für Sie in Ihrem Alltag ein Stockwerk Treppe ohne Geländer zu steigen?**

| (1) | (2) | (3) | (4) | (5) | (6) | (7) |
|---|---|---|---|---|---|---|
| ☐ | ☐ | ☐ | ☐ | ☐ | ☐ | ☐ |
| Gar nicht schwierig | sehr wenig schwierig | wenig schwierig | mässig schwierig | sehr schwierig | extrem schwierig | unmöglich |

**13) Wie schwierig ist es für Sie in Ihrem Alltag öffentliche Verkehrsmittel wie einen Bus, einen Zug oder ein Flugzeug zu benützen?**

| (1) | (2) | (3) | (4) | (5) | (6) | (7) |
|---|---|---|---|---|---|---|
| ☐ | ☐ | ☐ | ☐ | ☐ | ☐ | ☐ |
| Gar nicht schwierig | sehr wenig schwierig | wenig schwierig | mässig schwierig | sehr schwierig | extrem schwierig | unmöglich |

## Körperpflege und –hygiene

**14) Wie schwierig ist es für Sie in Ihrem Alltag Ihre Haare zu waschen?**

| (1) | (2) | (3) | (4) | (5) | (6) | (7) |
|---|---|---|---|---|---|---|
| ☐ | ☐ | ☐ | ☐ | ☐ | ☐ | ☐ |
| Gar nicht schwierig | sehr wenig schwierig | wenig schwierig | mässig schwierig | sehr schwierig | extrem schwierig | unmöglich |

**15) Wie schwierig ist es für Sie in Ihrem Alltag ein Bad in einer Badewanne zu nehmen?**

| (1) | (2) | (3) | (4) | (5) | (6) | (7) |
|---|---|---|---|---|---|---|
| ☐ | ☐ | ☐ | ☐ | ☐ | ☐ | ☐ |
| Gar nicht schwierig | sehr wenig schwierig | wenig schwierig | mässig schwierig | sehr schwierig | extrem schwierig | unmöglich |

**16) Wie schwierig ist es für Sie in Ihrem Alltag Ihre Haare zu kämmen oder zu föhnen?**

| (1) | (2) | (3) | (4) | (5) | (6) | (7) |
|---|---|---|---|---|---|---|
| ☐ | ☐ | ☐ | ☐ | ☐ | ☐ | ☐ |
| Gar nicht schwierig | sehr wenig schwierig | wenig schwierig | mässig schwierig | sehr schwierig | extrem schwierig | unmöglich |

**17) Wie schwierig ist es für Sie in Ihrem Alltag Ihre Zehennägel zu schneiden oder zu feilen?**

| (1) | (2) | (3) | (4) | (5) | (6) | (7) |
|---|---|---|---|---|---|---|
| ☐ | ☐ | ☐ | ☐ | ☐ | ☐ | ☐ |
| Gar nicht schwierig | sehr wenig schwierig | wenig schwierig | mässig schwierig | sehr schwierig | extrem schwierig | unmöglich |

**18) Wie schwierig ist es für Sie in Ihrem Alltag Ihren Rücken zu waschen (eventuell mit einer Rückenbürste)?**

| (1) | (2) | (3) | (4) | (5) | (6) | (7) |
|---|---|---|---|---|---|---|
| ☐ | ☐ | ☐ | ☐ | ☐ | ☐ | ☐ |
| Gar nicht schwierig | sehr wenig schwierig | wenig schwierig | mässig schwierig | sehr schwierig | extrem schwierig | unmöglich |

**19) Wie schwierig ist es für Sie in Ihrem Alltag sich nach dem Gang zur Toilette selbst zu reinigen?**

| (1) | (2) | (3) | (4) | (5) | (6) | (7) |
|---|---|---|---|---|---|---|
| ☐ | ☐ | ☐ | ☐ | ☐ | ☐ | ☐ |
| Gar nicht schwierig | sehr wenig schwierig | wenig schwierig | mässig schwierig | sehr schwierig | extrem schwierig | unmöglich |

**20) Wie schwierig ist es für Sie in Ihrem Alltag einen Pullover an- bzw. auszuziehen?**

| (1) | (2) | (3) | (4) | (5) | (6) | (7) |
|---|---|---|---|---|---|---|
| ☐ | ☐ | ☐ | ☐ | ☐ | ☐ | ☐ |
| Gar nicht schwierig | sehr wenig schwierig | wenig schwierig | mässig schwierig | sehr schwierig | extrem schwierig | unmöglich |

**21) Wie schwierig ist es für Sie in Ihrem Alltag Hosen oder Strumpfhosen an- bzw. auszuziehen?**

| (1) | (2) | (3) | (4) | (5) | (6) | (7) |
|---|---|---|---|---|---|---|
| ☐ | ☐ | ☐ | ☐ | ☐ | ☐ | ☐ |
| Gar nicht schwierig | sehr wenig schwierig | wenig schwierig | mässig schwierig | sehr schwierig | extrem schwierig | unmöglich |

**22) Wie schwierig ist es für Sie in Ihrem Alltag Schuhe oder Stiefel an- bzw. auszuziehen?**

| (1) | (2) | (3) | (4) | (5) | (6) | (7) |
|---|---|---|---|---|---|---|
| ☐ | ☐ | ☐ | ☐ | ☐ | ☐ | ☐ |
| Gar nicht schwierig | sehr wenig schwierig | wenig schwierig | mässig schwierig | sehr schwierig | extrem schwierig | unmöglich |

## Arbeiten im Haushalt

**23) Wie schwierig ist es für Sie in Ihrem Alltag tägliche Einkäufe nach Hause zu transportieren?**

| (1) | (2) | (3) | (4) | (5) | (6) | (7) |
|---|---|---|---|---|---|---|
| ☐ | ☐ | ☐ | ☐ | ☐ | ☐ | ☐ |
| Gar nicht schwierig | sehr wenig schwierig | wenig schwierig | mässig schwierig | sehr schwierig | extrem schwierig | unmöglich |

**24) Wie schwierig ist es für Sie in Ihrem Alltag Teller oder Gläser in den oberen Teil des Schrankes zu versorgen?**

| (1) | (2) | (3) | (4) | (5) | (6) | (7) |
|---|---|---|---|---|---|---|
| ☐ | ☐ | ☐ | ☐ | ☐ | ☐ | ☐ |
| Gar nicht schwierig | sehr wenig schwierig | wenig schwierig | mässig schwierig | sehr schwierig | extrem schwierig | unmöglich |

**25) Wie schwierig ist es für Sie in Ihrem Alltag den Boden nass aufzunehmen?**

| (1) | (2) | (3) | (4) | (5) | (6) | (7) |
|---|---|---|---|---|---|---|
| ☐ | ☐ | ☐ | ☐ | ☐ | ☐ | ☐ |
| Gar nicht schwierig | sehr wenig schwierig | wenig schwierig | mässig schwierig | sehr schwierig | extrem schwierig | unmöglich |

**26) Wie schwierig ist es für Sie in Ihrem Alltag zu staubsaugen?**

| (1) | (2) | (3) | (4) | (5) | (6) | (7) |
|---|---|---|---|---|---|---|
| ☐ | ☐ | ☐ | ☐ | ☐ | ☐ | ☐ |
| Gar nicht schwierig | sehr wenig schwierig | wenig schwierig | mässig schwierig | sehr schwierig | extrem schwierig | unmöglich |

**27) Wie schwierig ist es für Sie in Ihrem Alltag Ihr Haus und Garten oder Ihre Wohnung in Stand zu halten (beispielsweise Fenster zu putzen oder den Rasen zu mähen)?**

| (1) | (2) | (3) | (4) | (5) | (6) | (7) |
|---|---|---|---|---|---|---|
| ☐ | ☐ | ☐ | ☐ | ☐ | ☐ | ☐ |
| Gar nicht schwierig | sehr wenig schwierig | wenig schwierig | mässig schwierig | sehr schwierig | extrem schwierig | unmöglich |

**28) Wie schwierig ist es für Sie in Ihrem Alltag Haushaltsgeräte zu reinigen und in Stand zu halten (beispielsweise einen Staubsauger, einen Ofen oder elektrische Geräte)?**

| (1) | (2) | (3) | (4) | (5) | (6) | (7) |
|---|---|---|---|---|---|---|
| ☐ | ☐ | ☐ | ☐ | ☐ | ☐ | ☐ |
| Gar nicht schwierig | sehr wenig schwierig | wenig schwierig | mässig schwierig | sehr schwierig | extrem schwierig | unmöglich |

**29) Wie schwierig ist es für Sie in Ihrem Alltag den Kontakt mit Freunden und Verwandten aufrecht zu erhalten bzw. sie zu treffen?**

| (1) | (2) | (3) | (4) | (5) | (6) | (7) |
|---|---|---|---|---|---|---|
| ☐ | ☐ | ☐ | ☐ | ☐ | ☐ | ☐ |
| Gar nicht schwierig | sehr wenig schwierig | wenig schwierig | mässig schwierig | sehr schwierig | extrem schwierig | unmöglich |

**30) Wie schwierig ist es für Sie in Ihrem Alltag Überanstrengung zu vermeiden?**

| (1) | (2) | (3) | (4) | (5) | (6) | (7) |
|---|---|---|---|---|---|---|
| ☐ | ☐ | ☐ | ☐ | ☐ | ☐ | ☐ |
| Gar nicht schwierig | sehr wenig schwierig | wenig schwierig | mässig schwierig | sehr schwierig | extrem schwierig | unmöglich |

**31) Wie schwierig ist es für Sie in Ihrem Alltag ihre Arbeit, Schule und/oder Hausarbeit zu bewältigen?**

| (1) | (2) | (3) | (4) | (5) | (6) | (7) |
|---|---|---|---|---|---|---|
| ☐ | ☐ | ☐ | ☐ | ☐ | ☐ | ☐ |
| Gar nicht schwierig | sehr wenig schwierig | wenig schwierig | mässig schwierig | sehr schwierig | extrem schwierig | unmöglich |

**32) Wie schwierig ist es für Sie in Ihrem Alltag Freizeitaktivitäten, die Ihnen Spass machen, ausführen zu können?**

| (1) | (2) | (3) | (4) | (5) | (6) | (7) |
|---|---|---|---|---|---|---|
| ☐ | ☐ | ☐ | ☐ | ☐ | ☐ | ☐ |
| Gar nicht schwierig | sehr wenig schwierig | wenig schwierig | mässig schwierig | sehr schwierig | extrem schwierig | unmöglich |

**Besten Dank für das Ausfüllen des Fragebogens!**

**Auswertung des Myositis-Aktivitätenprofils**

Organisieren Sie alle Antworten in jeder Kategorie von 1-7, wobei 1 “überhaupt nicht schwierig” und 7 “unmöglich” ist

Kategorie „Bewegung“ mittlerer Wert = 4. Wert vom niedrigsten aus

Kategorie „Aktivitäten der Fortbewegung“ mittlerer Wert = 2. Wert vom niedrigsten aus

Kategorie „Körperpflege und Hygiene“ mittlerer Wert = 5. Wert vom niedrigsten aus

Kategorie „Arbeiten im Haushalt“ mittlerer Wert = 3. Wert vom niedrigsten aus

Die vier Einzelfragen über „soziale Aktivitäten“, „Vermeidung von Überanstrengung“, „Arbeit/Schule/Haushalt“ und „Erholung“ sind in keiner Kategorie enthalten und werden jeweils separat als Einzelkategorien gewertet.

Schreiben Sie den mittleren Wert sowohl jeder Kategorie als auch jeder Antwort von den Einzelfragen in die untenstehende Tabelle.

Mittlerer Wert = 1 bedeutet “überhaupt nicht schwierig” die Aktivität eines Abschnittes oder einer Einzelfrage auszuführen

Mittlerer Wert = 7 bedeutet “unmöglich” die Aktivität eines Abschnittes oder einer Einzelfrage auszuführen

| Datum | |
|---|---|
| Subskala/Einzelfrage | Mittlere Wert / Median |
| Bewegung | |
| Aktivitäten der Fortbewegung | |
| Körperpflege und -hygiene | |
| Arbeiten im Haushalt | |
| Soziale Aktiviäten | |
| Vermeidung on Überanstrengung | |
| Arbeit / Schule/Haushalt | |
| Erholung | |

**Name Untersucher/in:** **Unterschrift:**
